# Nurse Leaders’ Assertiveness as Perceived by Staff Nurses and Its Influence on Their Job Performance in Al‐Qassim Region Hospitals

**DOI:** 10.1155/jonm/9438410

**Published:** 2026-04-09

**Authors:** Ahmed Ibrahim Alshudukhi, Ahmed Mansour Almansour

**Affiliations:** ^1^ Training Centre, Prince Sultan Cardiac Center Qassim, Buraydah, Saudi Arabia; ^2^ Department of Advanced Nursing Care, College of Nursing, Majmaah University, Al-Majmaah, 11952, Saudi Arabia, mu.edu.sa

**Keywords:** assertiveness, job performance, nurses, Saudi Arabia

## Abstract

**Background:**

Assertiveness is recognized as a crucial leadership competency in nursing, linked to efficient communication, decision‐making, and patient safety. Previous studies have investigated assertiveness in nursing; however, there is a paucity of research on the impact of nurse leaders’ assertiveness on staff job performance, especially within the Saudi context, where healthcare reforms under Vision 2030 prioritize workforce leadership and development.

**Aim:**

The objective of this study was to evaluate the assertiveness of nurse leaders and investigate its correlation with the job performance of staff nurses in hospitals located in the Al‐Qassim region of Saudi Arabia.

**Methodology:**

The study utilized a cross‐sectional quantitative design. A total of 406 nurses from 17 hospitals were selected through convenience sampling for participation. Data were gathered utilizing two validated instruments: the Functional Assertiveness Scale (FAS) and the Job Performance Scale (JPS). Reliability was established, with FAS *α* = 0.962 and JPS *α* = 0.988. The analysis of the data used SPSS v26 to run descriptive statistics, *t*‐tests, ANOVA, Pearson correlations, and regression. It also gave effect sizes and confidence intervals.

**Results:**

The findings reveal that nurse leaders demonstrated a considerable degree of assertiveness (*M* = 4.43, SD = 0.81), while staff nurses indicated a significant level of job performance (*M* = 4.68, SD = 0.70). A notable positive correlation exists between assertiveness and job performance (*p* < 0.001). Both objective effectiveness and pragmatic politeness, as subdimensions of assertiveness, independently predicted job performance. Assertiveness and performance exhibited significant variation across demographic characteristics such as gender, nationality, and education.

**Conclusion:**

The assertiveness of nurse leaders has a significant impact on the job performance of staff, underscoring its relevance as both a theoretical concept and a practical skill.

## 1. Introduction

Nursing serves as an important component of healthcare delivery, with nurses constituting the largest segment of the global healthcare workforce. Well‐trained, empowered, and motivated nurses are essential for achieving universal health coverage, enhancing care quality, and ensuring patient safety [1, 2]. In Saudi Arabia, healthcare reforms under Vision 2030 aim to improve access and quality, making the role of nurse leaders in guiding and motivating staff increasingly essential [3, 4]. Behaviors of leaders such as communication, decision‐making, and assertiveness are consistently associated with organizational effectiveness and patient outcomes [5, 6].

Assertiveness is the capacity to articulate one’s thoughts, needs, and opinions with clarity, confidence, and respect, while also considering the perspectives of others [7]. In nursing, assertiveness enables professionals to advocate for patient safety, facilitate effective communication within multidisciplinary teams, and uphold ethical boundaries. Nurse leaders must balance authority and empathy while managing diverse teams in stressful healthcare environments [8, 9]. Consequently, an assertiveness training program may improve nurses’ psychological well‐being and work engagement [10].

Nursing job performance refers to the degree to which nurses fulfill their professional responsibilities effectively and efficiently, encompassing clinical competence, teamwork, adherence to standards, and innovation in practice [11]. Elevated job performance is crucial for the attainment of organizational objectives, the assurance of quality patient care, and the minimization of medical errors. Prior research indicates that the leadership styles and behavioral characteristics of nurse leaders, including empowerment, communication, and assertiveness, significantly influence staff performance [12–14].

A considerable amount of research demonstrates a connection between assertiveness and favorable nursing outcomes, such as increased self‐efficacy, reduced burnout, and improved collaboration [14–16]. However, evidence regarding the direct relationship between nurse leaders’ assertiveness and staff nurses’ job performance is limited, especially in Middle Eastern contexts. Within the Saudi context, many studies have examined nurse managers’ leadership styles and their effects on staff satisfaction, turnover intention, and nurses’ engagement [17, 18]. Another recent study has investigated the influence of nurse leaders’ behaviors on nurses [19]. However, to the best of our knowledge, no empirical study has specifically assessed how staff nurses perceive nurse leaders’ assertiveness and investigated its influence on their job performance. This study gap is particularly important given the ongoing healthcare transformation under Saudi Vision 2030, which emphasizes leadership enhancement and performance optimization within the healthcare workforce [3, 20–22].

Accordingly, this study aims to assess the level of assertiveness among nurse leaders, as perceived by staff nurses, and to examine its influence on nurses’ job performance in different hospitals within the Al‐Qassim region of Saudi Arabia. The hypothesis of the study posits that there is a statistically significant positive relationship between nurse leaders’ assertiveness and staff nurses’ job performance. By focusing on this gap, this study can contribute to the nursing leadership literature and provide empirical evidence linking nurse leaders’ assertiveness to nurses’ job performance. Hence, examining this relationship helps better understand assertiveness as a competence for nurse leaders and shows how it affects the performance of staff nurses.

## 2. Methodology

### 2.1. Study Design

This study utilized a cross‐sectional quantitative design, employing a self‐administered survey to evaluate the assertiveness of nurse leaders and its correlation with the job performance of staff nurses. A cross‐sectional design was selected because it enables researchers to measure both variables at a single point in time across a large sample, allowing for the examination of associations and group differences efficiently [23]. This design is particularly appropriate for workforce studies in healthcare, where timely assessments are necessary to inform leadership development, staffing policies, and organizational performance initiatives.

### 2.2. Settings

This study was carried out in 17 hospitals within the Al‐Qassim Health Cluster, which is in the Al‐Qassim region of Saudi Arabia. The region is in the middle of the country and includes both cities, like Buraydah and Unaizah, and rural areas that provide healthcare to smaller towns and villages [24]. The 17 different hospitals were as follows: 1 tertiary hospital, 2 specialized centers, and 14 general hospitals, representing different levels of healthcare settings within the cluster. The cluster’s hospitals have a capacity of more than 2900 beds [24]. The hospitals serve a wide range of people from different social and economic backgrounds, including both Saudi citizens and foreigners. Patients come from a wide range of backgrounds. Some live in rural areas and come from middle‐ and lower‐income families, while others live in urban areas and come from middle‐ to upper‐income families. This variety is a good representation of the Saudi nursing workforce and the population they provide care for. The inclusion of hospitals with different healthcare levels can strengthen the generalizability of the study findings across different healthcare contexts [24].

### 2.3. Sampling and Participants

The study sample comprised registered nurses employed in hospitals affiliated with the Al‐Qassim Health Cluster, Saudi Arabia. Participants were recruited from various hospitals, encompassing both tertiary referral centers and general hospitals, thereby ensuring diversity in practice environments, patient demographics, and service provision. Participants were recruited using convenience sampling. The sample size calculation was based on the total number of staff nurses employed in the cluster hospitals. The approximate number of nurses employed is more than 5000 nurses in the cluster’s different hospitals, as per the latest reported number [24]. Using the Raosoft sample size calculator, to ensure representativeness with a 5% margin of error and 95% confidence level, the minimum sample size required was 357 staff nurses [25]. The inclusion criteria were registered nurses employed in Al‐Qassim region hospitals who have at least 1 year of experience as staff nurses and are willing to participate in this study. Those in leadership positions, have less than 1 year of experience, and are not willing to participate were excluded. The final participants recruited in this study were 406 staff nurses employed across different hospitals.

### 2.4. Measurement Tools

The questionnaire utilized for this study contained three parts. The first part contained sociodemographic characteristics of the participants, such as age, gender, nationality, marital status, and educational level. The second part contained the Functional Assertiveness Scale (FAS), developed by Mitamura in 2018, which was utilized to assess the level of nurse leaders’ assertiveness from nurses’ perceptions [26]. The scale is comprised of 12 items, scored on a 5‐point Likert‐type scale, ranging from 1 (*always disagree*) to 5 (*always agree*) for each item. The FAS comprises two subscales. The first subscale, objective effectiveness, assesses the capacity to manage interpersonal conflicts or issues independently. The second subscale, pragmatic politeness, evaluates the ability to effectively manage interpersonal disagreements or issues. Cronbach’s alpha reported for the FAS was 0.84, and the scoring system of the scale ranges from 12 to 60, with elevated scores indicating an increased degree of assertiveness [26]. The third part of the questionnaire contained the Job Performance Scale (JPS), developed by de Azevedo Andrade et al. in 2020, which was used to assess the staff nurses’ job performance [27]. The scale is comprised of 10 items, scored on a 5‐point Likert‐type scale, ranging from 1 (*strongly disagree*) to 5 (*strongly agree*) for each item. The JPS comprises two subscales. The first subscale, task performance, assesses the skills the individual learns to perform a task or develop a product. The second subscale, contextual performance, assesses the idea of organizational behavior and the commitment to the organization. Cronbach’s alpha reported for the JPS was 0.85, and the scoring system of the scale ranges from 10 to 50, with elevated scores indicating a high level of job performance [27].

### 2.5. Validity and Reliability

The tools used in this study have been extensively employed in healthcare research and have exhibited robust psychometric properties across various populations [28, 29]. The tools were also reviewed by a panel of three experts in nursing leadership to ensure their appropriateness for this study. In addition, the reliability of both tools was tested using Cronbach’s alpha. In this study, the FAS demonstrated a Cronbach’s alpha of 0.962, and the JPS demonstrated a Cronbach’s alpha of 0.988, indicating excellent internal consistency. These values were higher than those reported in previous validation studies (FAS: *α* = 0.85; JPS: *α* = 0.89–0.92), confirming the robustness of the tools in this study’s context.

### 2.6. Data Collection

To collect data, the researchers got in touch with the nursing administration offices at hospitals to ask for permission and help with the study. Once approval was granted, an invitation containing study information with a link to the online questionnaire was circulated via official hospital email lists and WhatsApp groups. The nursing supervisors were asked to share the survey invitation with eligible nurses working under their supervision. Prior to accessing the questionnaire, all participants were presented with an electronic informed consent form on the first page of the survey. This form explained the study’s purpose, procedures, voluntary nature of participation, expected time to complete the questionnaire, and potential benefits and minimal risks. Participants were explicitly informed that they could withdraw at any stage before submitting the survey without providing a reason and without any consequences. Only those who clicked the “I agree to participate” option were granted access to the survey questions. The questionnaire was conducted online using Google Forms. This method was chosen to make it easier for nurses to participate across the multiple hospitals, minimize disruptions to clinical duties, and ensure that the participants could complete the survey at a convenient time. On average, the survey took approximately 10–15 min to complete. The data collection was conducted between February and March 2025 across the participating hospitals within the Al‐Qassim Health Cluster.

### 2.7. Ethical Considerations

This study was conducted in accordance with the ethical principles outlined in the Declaration of Helsinki and institutional research guidelines. Approval to conduct the research was obtained from the Institutional Review Board (IRB) of the Al‐Qassim Health Cluster (IRB Log No.: 607‐46‐010269), as well as from the participating hospitals. All participants were provided with an electronic information sheet describing the study’s purpose, procedures, expected time commitment, potential risks, and anticipated benefits. They were informed that participation was entirely voluntary and that they could decline or withdraw from the study at any time without penalty or impact on their employment. Informed consent was obtained electronically before participants could access the survey questions, and only those who provided consent were included in the study. To ensure anonymity and confidentiality, no identifying information (e.g., names, staff numbers, or contact details) was collected. The online questionnaire was administered through a secure platform (Google Forms) that did not track IP addresses. Data were stored in a password‐protected database accessible only to the research team. Findings are reported in aggregate form, preventing identification of individual participants or specific hospitals.

### 2.8. Data Analysis

The statistical analysis was performed with IBM SPSS Statistics Version 27. The analysis involved the use of various statistical techniques to test the research hypothesis. Descriptive statistics, including frequencies and percentages, were computed to characterize the demographic profile of the nurse participants. The internal consistency of the measurement scales was evaluated using Cronbach’s alpha, with a threshold of 0.7. Continuous variables were summarized using means and standard deviations. To explore differences based on demographic factors, independent sample *t*‐tests and one‐way ANOVA were performed, followed by post hoc Tukey’s HSD tests for detailed pairwise comparisons when necessary. Furthermore, Pearson correlation analysis was applied to assess the relationship between the level of nurse leaders’ assertiveness and staff nurses’ job performance.

## 3. Results

### 3.1. Participants’ Characteristics

Table 1 presents the sociodemographic characteristics of the study participants (*N* = 406). They were predominantly male (75.4%), single (48.3%), and fell within the 20–30 year age group (40.6%). The vast majority were Saudi nationals (78.6%) and held a bachelor’s degree (86.9%). The most frequently reported years of experience were 1–5 years (31.5%).

**TABLE 1:** Descriptive statistics of the sociodemographic characteristics (*N* = 406).

Characteristics	Categories	*N*	%
Age (years)	20–30	165	40.6
	31–40	147	36.2
	41–50	89	21.9
	≥ 51	5	1.2

Gender	Male	306	75.4
	Female	100	24.6

Nationality	Saudi	319	78.6
	Non‐Saudi	87	21.4

Marital status	Single	196	48.3
	Married	195	48.0
	Divorced	13	3.2
	Widowed	2	0.5

Educational level	Diploma	34	8.4
	Bachelor	353	86.9
	Master or higher	19	4.7

Years of experience	1–5	128	31.5
	6–10	80	19.7
	11–15	104	25.6
	16–20	54	13.3
	> 20	40	9.9

### 3.2. Descriptive Statistics of the Study Variables

Table 2 presents the means and standard deviations for nurse leader assertiveness among the participating nurses. On a maximum score of 5, the total mean score on the nurse leader assertiveness scale was 4.43 (SD = 0.81), reflecting a high level of perceived assertiveness. Among the subscales, the objective effectiveness subscale received the highest score (*M* = 4.44, SD = 0.79), followed closely by the pragmatic politeness subscale (*M* = 4.41, SD = 0.86).

**TABLE 2:** Means and standard deviations of the Functional Assertiveness Scale (FAS).

Subscale	Mean ± SD
Objective effectiveness subscale	4.44 ± 0.79
Pragmatic politeness subscale	4.41 ± 0.86
Total Functional Assertiveness Scale	4.43 ± 0.81

Table 3 demonstrates the means and standard deviations of the objective effectiveness subscale items from the nurse leader assertiveness scale. The findings show that the highest mean score was observed for the item “The manager can get a teammate or a peer to change my behavior if I am being disruptive” (*M* = 4.52, SD = 0.98). This was closely followed by “The manager can get me to adjust my way of dressing if he feels that my appearance is not OK” (*M* = 4.47, SD = 0.91). Meanwhile, the lowest mean score was observed for the item “The manager can get a person to understand that I am being unjust if I point out his failures due to a misunderstanding” (*M* = 4.35, SD = 0.99).

**TABLE 3:** Means and standard deviations of the objective effectiveness subscale.

Item	Mean ± SD
1. The manager can get a teammate or a peer to change my behavior if I am being disruptive.	4.52 ± 0.98
2. The manager can get people to understand his own ideas even if his ideas are different from theirs.	4.38 ± 0.94
3. The manager can get me to improve my manners if he feels my manners are not OK.	4.46 ± 0.88
4. The manager can get me to adjust my way of dressing if he feels that my appearance is not OK.	4.47 ± 0.91
5. The manager can get a person to understand that I am being unjust if I point out his failures due to a misunderstanding.	4.35 ± 0.99
6. The manager can get his friends to stop their annoying and troublesome actions.	4.46 ± 0.95

Table 4 illustrates the means and standard deviations of the pragmatic politeness subscale items from the nurse leader assertiveness scale. According to this table, the highest mean score was observed for the item “The manager doesn’t needlessly embarrass someone when he tries to get that person to improve his/her manners” (*M* = 4.47, SD = 0.91). This was followed by two items: “The manager never makes people feel bad when he tries to get them to understand his own ideas” (*M* = 4.44, SD = 0.91) and “The manager doesn’t carelessly insult my friend when he tries to get him/her to stop annoying or troublesome actions” (*M* = 4.44, SD = 0.96). Meanwhile, the lowest mean score was observed for the item “The manager doesn’t get on a person’s nerves more than necessary when he tries to get the person to understand that he/she is being unjust in pointing out his failures” (*M* = 4.34, SD = 1.07).

**TABLE 4:** Means and standard deviations of the pragmatic politeness subscale.

Item	Mean ± SD
7. The manager doesn’t offend a disruptive teammate or a peer when he tries to get that person to change his/her behavior.	4.40 ± 1.00
8. The manager never makes people feel bad when he tries to get them to understand his own ideas.	4.44 ± 0.91
9. The manager doesn’t needlessly embarrass someone when he tries to get that person to improve his/her manners.	4.47 ± 0.91
10. The manager causes minimal embarrassment to a person when he tries to get that person to change the way he/she dresses.	4.39 ± 1.00
11. The manager doesn’t get on a person’s nerves more than necessary when he tries to get the person to understand that he/she is being unjust in pointing out his failures.	4.34 ± 1.07
12. The manager doesn’t carelessly insult my friend when he tries to get him/her to stop annoying or troublesome actions.	4.44 ± 0.96

Table 5 presents the means and standard deviations for job performance among the participating nurses. On a maximum score of 5, the total mean score on the JPS was 4.68 (SD = 0.70), reflecting a very high level of self‐reported performance. In relation to subscales, the context subscale received a nearly identical score (*M* = 4.68, SD = 0.70) to the task subscale (*M* = 4.67, SD = 0.71), indicating that nurses perceived themselves as highly proficient in both their core duties and their contextual, proactive work behaviors.

**TABLE 5:** Means and standard deviations of the Job Performance Scale (JPS).

Subscale	Mean ± SD
Task subscale	4.67 ± 0.71
Context subscale	4.68 ± 0.70
Total Job Performance Scale	4.68 ± 0.70

Table 6 reveals the means and standard deviations of the task performance subscale items from the JPS. According to this table, the highest mean scores were observed for the items “I perform hard tasks properly” and “I do my job according to what the organization expects from me” (both *M* = 4.68, SD = 0.73 and 0.74, respectively). Meanwhile, the lowest mean score was observed for the item “I plan the execution of my job by defining actions, deadlines and priorities” (*M* = 4.65, SD = 0.76).

**TABLE 6:** Means and standard deviations of the task subscale.

Item	Mean ± SD
1. I perform hard tasks properly.	4.68 ± 0.73
2. I do my job according to what the organization expects from me.	4.68 ± 0.74
3. I plan the execution of my job by defining actions, deadlines, and priorities.	4.65 ± 0.76
4. I plan actions according to my tasks and organizational routines.	4.67 ± 0.75
5. I seize opportunities that can improve my results at work.	4.67 ± 0.77

Table 7 illustrates the means and standard deviations of the contextual performance subscale items from the JPS. According to this table, the highest mean score was observed for the item “I work hard to do the tasks designated to me” (*M* = 4.70, SD = 0.71). This was closely followed by the item “I try to update my technical knowledge to do my job” (*M* = 4.69, SD = 0.72). Meanwhile, the lowest mean score was observed for the item “I seek new solutions for problems that may come up in my job” (*M* = 4.67, SD = 0.75).

**TABLE 7:** Means and standard deviations of the context subscale.

Item	Mean ± SD
6. I try to update my technical knowledge to do my job.	4.69 ± 0.72
7. I take initiatives to improve my results at work.	4.68 ± 0.72
8. I seek new solutions for problems that may come up in my job.	4.67 ± 0.75
9. I work hard to do the tasks designated to me.	4.70 ± 0.71
10. I execute my tasks foreseeing their results.	4.68 ± 0.74

### 3.3. Differences in Nurse Leader Assertiveness Perception and Job Performance Based on Demographic Characteristics

Table 8 outlines the differences in nurse leader assertiveness perception among the studied nurses based on various demographic characteristics. The results showed that there were statistically significant differences in perceived assertiveness based on all tested demographics.

**TABLE 8:** Differences in nurse leader assertiveness among studied nurses based on their characteristics (*N* = 406).

Characteristics		Mean ± SD	Test of significance	*p*
Age (years)	20–30^a^	4.64 ± 0.71	*F* = 6.464	< 0.001 a > b, c, d
	31–40^b^	4.28 ± 0.78		
	41–50^c^	4.29 ± 0.93		
	≥ 51^d^	4.20 ± 0.83		

Gender	Male	4.65 ± 0.60	*t* = 11.386	< 0.001
	Female	3.73 ± 0.95		

Nationality	Saudi	4.65 ± 0.61	*t* = 12.386	< 0.001
	Non‐Saudi	3.62 ± 0.92		

Marital status	Single^a^	4.64 ± 0.65	*F* = 9.995	< 0.001 a > b
	Married^b^	4.22 ± 0.90		
	Divorced^c^	4.39 ± 0.66		
	Widowed^d^	4.00 ± 1.41		

Education	Diploma^a^	4.05 ± 0.88	*F* = 14.891	< 0.001 b > a, c
	Bachelor^b^	4.50 ± 0.76		
	Master or higher^c^	3.66 ± 1.01		

Work experience in nursing	1–5^a^	4.55 ± 0.83	*F* = 2.402	0.049 a > e
	6–10^b^	4.37 ± 0.79		
	11–15^c^	4.48 ± 0.69		
	16–20^d^	4.35 ± 0.84		
	> 20^e^	4.14 ± 0.93		

*Note:* *F* = one‐way analysis of variance; *t* = independent sample *t*‐test.

^abcde^Differences between the means by Tukeys’ HSD post hoc test.

The one‐way analysis of variance and *t*‐tests revealed significant differences in perceived assertiveness by age (*F* = 6.464, *p* < 0.001), gender (*t* = 11.386, *p* < 0.001), nationality (*t* = 12.386, *p* < 0.001), marital status (*F* = 9.995, *p* < 0.001), and education level (*F* = 14.891, *p* < 0.001). Post hoc analyses indicated that nurses aged 20–30 years perceived significantly higher assertiveness than all other age groups. Single nurses reported higher scores than their married colleagues, and those with bachelor’s degrees reported higher scores than those with diplomas or master’s degrees. Finally, the overall ANOVA for years of experience showed a marginally significant difference (*F* = 2.402, *p* = 0.049), with post hoc tests indicating that nurses with 1–5 years of experience reported significantly higher perceived assertiveness than those with over 20 years of experience.

Table 9 outlines the differences in job performance among the studied nurses based on various demographic characteristics. The results showed that there were statistically significant differences in job performance based on all tested demographics.

**TABLE 9:** Differences in job performance among studied nurses based on their characteristics (*N* = 406).

Characteristics		Mean ± SD	Test of significance	*p*
Age (years)	20–30^a^	4.82 ± 0.44	*F* = 3.758	0.011 a > c
	31–40^b^	4.59 ± 0.73		
	41–50^c^	4.57 ± 0.96		
	≥ 51^d^	4.60 ± 0.89		

Gender	Male	4.83 ± 0.47	*t* = 8.417	< 0.001
	Female	4.21 ± 1.02		

Nationality	Saudi	4.82 ± 0.49	*t* = 8.601	< 0.001
	Non‐Saudi	4.15 ± 1.03		

Marital status	Single^a^	4.81 ± 0.44	*F* = 5.106	0.002 a > d
	Married^b^	4.56 ± 0.86		
	Divorced^c^	4.56 ± 0.84		
	Widowed^d^	4.00 ± 1.41		

Education	Diploma^a^	4.38 ± 0.76	*F* = 8.753	< 0.001 b > a, c
	Bachelor^b^	4.73 ± 0.64		
	Master or higher^c^	4.21 ± 1.21		

Work experience in nursing	1–5^a^	4.74 ± 0.53	*F* = 3.708	0.006 a, b, c > e
	6–10^b^	4.75 ± 0.45		
	11–15^c^	4.72 ± 0.58		
	16–20^d^	4.60 ± 0.95		
	> 20^e^	4.31 ± 1.23		

*Note: F* = one‐way analysis of variance; *t* = independent sample *t*‐test.

^abcde^Differences between the means by Tukeys’ HSD post hoc test.

The findings revealed significant differences in job performance by age (*F* = 3.758, *p* = 0.011), gender (*t* = 8.417, *p* < 0.001), nationality (*t* = 8.601, *p* < 0.001), marital status (*F* = 5.106, *p* = 0.002), education level (*F* = 8.753, *p* < 0.001), and years of experience (*F* = 3.708, *p* = 0.006). Post hoc analyses indicated that younger nurses (20–30 years) demonstrated significantly higher job performance than those aged 41–50 years. Single nurses showed higher performance than widowed nurses, and those with bachelor’s degrees reported better performance than those with diplomas or master’s degrees. Additionally, post hoc tests for years of experience indicated that nurses with 1–5 years, 6–10 years, and 11–15 years of experience all reported significantly higher job performance than those with over 20 years of experience (*F* = 3.708, *p* = 0.006).

### 3.4. Correlations Between Nurse Leader Assertiveness Perception Among the Studied Nurses and Their Job Performance

Table 10 illustrates the correlations between studied nurses’ perception of nurse leader assertiveness and their job performance. The analysis reveals significant positive correlations between the total nurse leader assertiveness score and job performance (*r* = 0.704, *p* < 0.001; large effect size). Additionally, both subscales of nurse leader assertiveness demonstrated strong positive correlations with job performance: objective effectiveness (*r* = 0.702, *p* < 0.001; large effect size) and pragmatic politeness (*r* = 0.674, *p* < 0.001; large effect size).

**TABLE 10:** Correlations between nurse leader assertiveness perception and job performance (*N* = 406).

Variable	Job performance *r* (*p*)
‐ Objective effectiveness subscale	0.702 (*p* < 0.001)
‐ Pragmatic politeness subscale	0.674 (*p* < 0.001)
Total nurse leader assertiveness perception	0.704 (*p* < 0.001)

## 4. Discussion

The objective of this study was to examine the perceived assertiveness of nurse leaders as evaluated by staff nurses and its relationship with job performance in hospitals located in the Al‐Qassim region of Saudi Arabia. The findings indicated that assertiveness and job performance were consistently high, demonstrating a notable prevalence of leadership behaviors and effective performance within this healthcare environment. The research demonstrated a notable correlation between increased assertiveness in nurse leaders and enhanced job performance among staff nurses, suggesting that assertiveness is essential for fostering effective teamwork, accountability, and productivity in clinical settings. Variations were observed in demographic factors, with significant differences in assertiveness and performance based on gender, nationality, marital status, age, and education. The findings highlight the intricate relationships between individual and professional traits that affect leadership behaviors and workplace performance. The findings emphasize the need to treat assertiveness as a fundamental leadership competency that directly impacts nursing outcomes and organizational effectiveness.

The finding that nurse leaders in Al‐Qassim hospitals demonstrated high levels of assertiveness aligns with existing literature that identifies assertiveness as an essential leadership trait in nursing practice [7, 9]. The job performance, as rated by staff nurses, was high, consistent with a study that evaluated job performance and its associated factors [30]. The findings of this study further establish a direct connection between assertiveness and enhanced staff job performance, thereby supporting the assertion that leadership communication behaviors are essential for organizational success. This association aligns with the findings of Gunawan et al., who demonstrated that leadership characteristics significantly affect staff motivation and performance [31]. This aligns with the research conducted by Alluhaybi et al., which emphasized the beneficial effects of nurse managers’ leadership styles on staff outcomes in Saudi hospitals [18]. The study demonstrated significantly elevated assertiveness and performance scores in contrast to findings from other contexts that indicated moderate levels of both [32, 33]. This suggests cultural and systemic variations; for example, the Saudi Vision 2030 reforms prioritize workforce development and leadership training, which may result in improved leadership behaviors and performance outcomes in this context.

The observed demographic variations, including increased assertiveness and job performance among male and Saudi nurses, align with previous research findings. Alsadaan et al. identified disparities in gender and nationality within the Saudi nursing workforce, which may affect professional confidence and the expression of leadership [34]. The findings suggest that these differences may lead to measurable variations in performance, constituting a significant contribution. This highlights the need for leadership training programs that address potential inequities and improve assertiveness skills among diverse staff groups. The two subdimensions of assertiveness, objective effectiveness, and pragmatic politeness, independently predicted job performance. This highlights the multifaceted nature of assertiveness as identified by Mitamura, suggesting that effective nurse leaders combine clarity and firmness with respect and diplomacy [26]. This insight contributes to the leadership literature by emphasizing that assertiveness consists of a set of skills that, when combined, can enhance performance outcomes. Our findings corroborate and expand upon existing research regarding the importance of leadership in nursing, while offering new insights into assertiveness as a unique leadership competency within the Saudi healthcare context.

This study adds to the existing literature on leadership theory in nursing by providing real‐world evidence that assertiveness is both a useful and measurable interpersonal leadership skill that has a direct impact on individual performance. Our research demonstrates that both objective effectiveness and pragmatic politeness substantially influence job performance, thereby endorsing multidimensional models of leadership that posit that effective leaders incorporate clarity, decisiveness, and respect in their communication [26]. The results show that guiding nurse leaders to be more assertive is important for improving staff performance and, as a result, the quality of patient care. Nurse leaders who communicate effectively, establish clear expectations, and handle conflicts with respect are more likely to promote teamwork, accountability, and professional development within their teams. The findings correspond with the healthcare reforms outlined in Saudi Vision 2030, which prioritize the development of leadership capabilities and the localization of the workforce. This study demonstrates a strong correlation between assertiveness and performance, providing evidence for the integration of assertiveness and communication skills training into continuing professional development frameworks and hospital accreditation standards. In nursing education, the integration of assertiveness and communication modules into both undergraduate and postgraduate curricula is essential for equipping future nurses with the necessary skills for leadership roles.

This study’s results reinforce theoretical advancements by framing assertiveness as a multidimensional leadership competency. They, as well, help to shape practice and policy by emphasizing its significance in workforce advancement, organizational performance, and healthcare transformation. Future research should conduct longitudinal studies to ascertain the causal relationship between assertiveness and performance. Interventional studies are essential to evaluate the efficacy of assertiveness training programs. Cross‐cultural comparisons can also be used to examine the effect of cultural norms on the role of assertiveness in leadership.

This study has several strengths that enhance the reliability and credibility of its findings. The study utilized a strong methodological framework, incorporating two validated instruments—the FAS and the JPS—which exhibited high reliability (*α* > 0.95) in this context. The study included a substantial and diverse sample of 406 nurses from 17 hospitals within the Al‐Qassim Health Cluster, which included a tertiary hospital, specialized centers, and general hospitals. This variety makes the results more representative and generalizable. The research provided a unique perspective by examining the assertiveness of nurse leaders as a predictor of staff job performance, thereby addressing a gap in both Saudi and international literature. The study’s contextual relevance to the Saudi Vision 2030 reforms enhances its implications for policy and practice, rendering the findings timely and significant for nursing leadership and workforce development.

The study, however, has some limitations. The cross‐sectional design constrains causal inference, suggesting that while associations were recognized, conclusive cause‐and‐effect relationships cannot be determined. The data collection utilized self‐reported questionnaires, which may result in response or social desirability bias. The sample, although substantial and varied within the Al‐Qassim Health Cluster, may not be fully generalizable to other regions of Saudi Arabia or elsewhere. In addition, the majority of the study’s sample were male nurses, which contradict the fact that many nurses are actually females in Saudi Arabia. This might be a result of more willingness of the male nurses to participate in this study. Finally, the absence of longitudinal or interventional data limits the evaluation of changes in assertiveness and job performance over time, as well as the assessment of the impacts of specific leadership training programs.

## 5. Conclusion

The aim of this study is to investigate the association between the assertiveness of nurse leaders and the staff nurses’ job performance in Al‐Qassim region hospitals, Saudi Arabia. The results showed that nurse leaders were consistently more assertive, and this was strongly linked to better staff performance. The two subdimensions of assertiveness—objective effectiveness and pragmatic politeness—demonstrated a positive impact on performance, pointing out the importance of balanced, respectful, and confident communication in leadership.

The findings enhance the theoretical framework of nursing leadership by identifying assertiveness as a multifaceted competency that improves performance outcomes. They highlight the necessity of assertiveness training and leadership development programs centered on communication within healthcare institutions. The findings advocate for the incorporation of leadership competencies, including assertiveness, into national workforce development strategies in accordance with Saudi Vision 2030.

The study presents evidence that assertiveness functions as both a personal trait and a professional skill, significantly influencing the effectiveness of nursing teams. Subsequent research ought to expand upon these findings, utilizing longitudinal and interventional methodologies, while considering cultural contexts and approaches for incorporating assertiveness into nursing education and practice.

## Funding

The author extends the appreciation to the Deanship of Postgraduate Studies and Scientific Research at Majmaah University for funding this research work through the project number (R‐2026‐105).

## Conflicts of Interest

The authors declare no conflicts of interest.

## Data Availability

The data that support the findings of this study are available from the corresponding author upon reasonable request.
